# Assessment of precision and reliability of a novel computerized heterophoria test

**DOI:** 10.3389/fnins.2023.1207945

**Published:** 2023-06-12

**Authors:** Yuwen Wang, Fuhao Zheng, Fengchao Zhou, E. Song

**Affiliations:** ^1^Department of Ophthalmology, the Second Affiliated Hospital of Soochow University, Suzhou, Jiangsu, China; ^2^Eye Hospital and School of Ophthalmology and Optometry, Wenzhou Medical University, Wenzhou, Zhejiang, China; ^3^Department of Ophthalmology, Lixiang Eye Hospital of Soochow University, Suzhou, Jiangsu, China

**Keywords:** heterophoria, repeatability, reliability, agreement, cover test, heterophoria method

## Abstract

**Purpose:**

To assess the precision and reliability of a novel computerized heterophoria test (CHT).

**Methods:**

One hundred and three subjects aged 20 to 48 (27.37 ± 5.15) were recruited from Wenzhou Medical University. All subjects with corrected spectacles were examined with CHT and a prism-neutralized objective cover test (POCT) in a randomized order. They were then re-examined with CHT within 1 week. Their heterophoria was measured at three different distances (3 m, 0.77 m and 0.4 m); the average was recorded after three consecutive measurements. Inter-examiner repeatability, intra-examiner repeatability of CHT and agreement between CHT and POCT were evaluated.

**Results:**

There was no significant difference among repeated measurements using CHT (all *p* > 0.05). The difference between POCT and CHT was statistically significant at three distances (all *p* < 0.001). However, the mean absolute difference was 1.20^△^, 1.93^△^, and 2.41^△^, all of which were significantly smaller than the permissible range of error (4^△^) at three different distances (all *p* < 0.001).

**Conclusion:**

The CHT demonstrated excellent inter- and intra-examiner repeatability, as well as good correlation with POCT. The differences between CHT and POCT were within the permissible range of error, indicating that CHT could provide a precise and reliable measurement for clinical applications.

## Introduction

Visual fatigue is a global health concern, with a high prevalence among younger populations and university students worldwide. Studies have reported that 12.4–32.2% of individuals below 18 years of age (Ip et al., 2006; Tiwari, 2013) and 46–71% of university students suffer from visual fatigue (Han et al., 2013; Hashemi et al., 2019). Binocular vision anomaly has been identified as a leading cause of visual fatigue (Scheiman and Wick, 2014), and studies have suggested a correlation between binocular vision anomaly and visual fatigue (Garcia-Munoz et al., 2014; Sheppard and Wolffsohn, 2018; Zheng et al., 2021). Measuring heterophoria is crucial in evaluating binocular vision anomaly, as phoria at near and far distances can assist in the differential diagnosis of various types of binocular vision anomaly (Scheiman and Wick, 2014).

Many individuals experience visual fatigue due to prolonged use of digital devices, such as computers, for reading and writing purposes (Carmichael, 1947; Argiles et al., 2016). Some studies have suggested that the viewing distance during near work may vary depending on the nature of different tasks (Eastwood-Sutherland and Gale, 2011; Argiles et al., 2016). For example, a study that employed an ultrasound sensor device found that the viewing distances for game, text completion, and web search tasks were approximately 54.5 cm, which was shorter than that of a video task (62.3 cm) (Argiles et al., 2016). Similarly, the viewing distances for reading and writing tasks can also differ depending on the task and age, ranging from 25 cm to 40 cm (Yeo et al., 2013; Boccardo, 2021). Therefore, assessing heterophoria at different distances can provide valuable insights into the binocular vision anomalies of patients.

Several methods have been utilized to evaluate heterophoria, including the prism-neutralized objective cover test (POCT), the modified Thorington test (TH), and the Maddox rod test (MR). POCT is often regarded as a reliable test (Rainey et al., 1998a,b; Johns et al., 2004). However, its accuracy may be influenced by the examiner’s experience, which introduces an external source of variance (Anderson et al., 2010; Hrynchak et al., 2010). TH is a popular method due to its simplicity (Schroeder et al., 1996; Cebrian et al., 2014). Nevertheless, the range of heterophoria and the measuring distance are limited by the length of the testing card (Cebrian et al., 2014). While MR allows for the measurement of heterophoria at any distance, its reliability and repeatability have been found to be inadequate (Rainey et al., 1998a,b; Cebrian et al., 2014).

To overcome the disadvantages of TH and MR, we developed the Computerized Heterophoria Test (CHT), which is based on the principle of TH and incorporates the advantages of MR. Heterophoria was measured by using Maddox rod to separate visual fields of two eyes and to scale the amplitude of heterophoria in the principle of TH. The goal of the study was to evaluate the reliability of CHT at different distances and its agreement with POCT, which is one of most popular tests for measuring heterophoria.

## Methods

### Subjects

The study recruited a total of 103 subjects (26 males and 77 females) from Wenzhou Medical University. All subjects underwent a comprehensive ophthalmic examination. Exclusion criteria included the history of eye surgery, trauma, strabismus, amblyopia, or any physical or mental impairment that could affect the test results. Subjects’ refractive error ranged from +1.00 D to −6.00 D, astigmatism was less than −1.00 D, anisometropia was less than 1.00 D, and the best-corrected visual acuity was not worse than 20/20. The study adhered to the tenets of the Declaration of Helsinki and was approved by the Research Ethics Committee at Eye Hospital of Wenzhou Medical University. Each subject provided written informed consent.

### Test methods

Heterophoria at three different distances were quantified using the CHT and POCT. Three distances were chosen based on clinical practice: 3 m, 0.77 m, and 0.40 m. The distance of 0.77 m (1.3D) represents an intermediate distance, as calculated by the average of the diopter of the distance (10 m, 0.1D) and near (0.4 m, 2.5D) based on Shibata’s study (Shibata et al., 2011). Heterophoria was performed in two sessions, with an interval of less than 1 week between sessions.

### Computerized heterophoria test

#### Equipment

**Figure 1:**
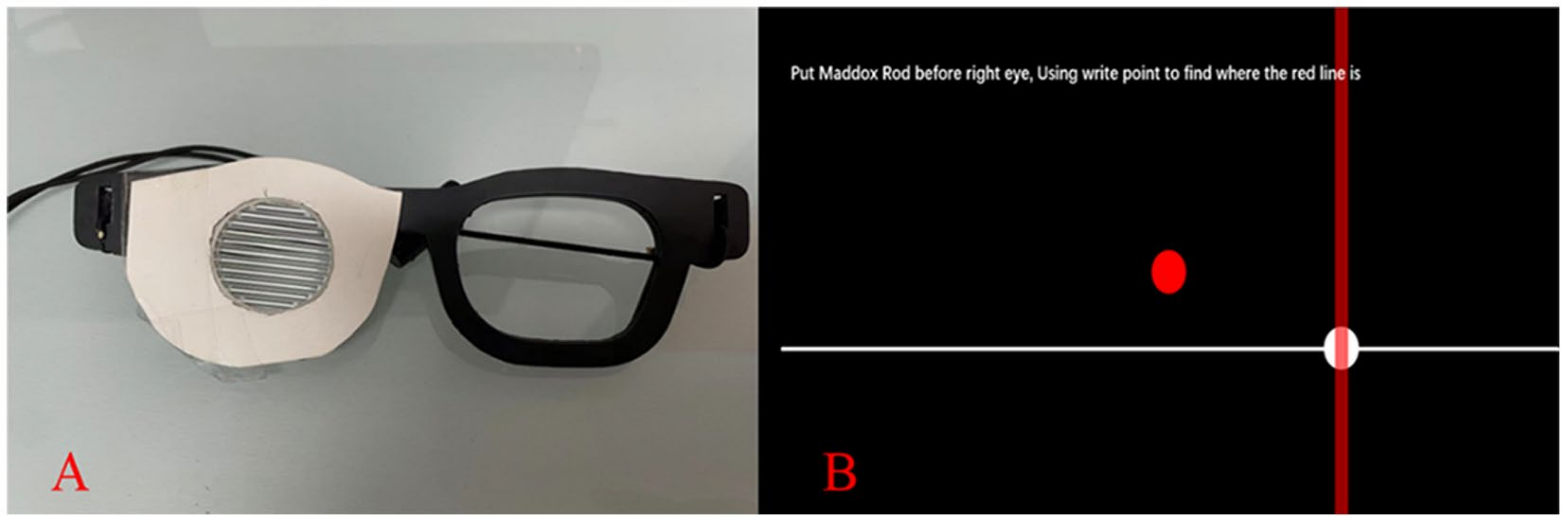
**(A)** An examinational spectacle with a horizontal Maddox rod on the right eye; **(B)** A schematic diagram of CHT. Subjects were asked to move the white point with the mouse to click the cross of red vertical line and white horizontal line. At 0.40-meter measurement, subjects were asked to keep the sentence clear during the test so that their accommodation would be stable.

The CHT was performed by a program which was written in MATLAB (MathWorks, Natick, MA) with PsychToolBox extensions (Kleiner et al., 2007). Stimuli were displayed on a 27-inch screen (ROG PG278QR, ASUS, Taiwan, China). The display had a spatial resolution of 2,560 × 1,440 pixels and a refresh rate of 60 Hz. An examinational spectacle with a white Maddox rod on right eye was used for examination (shown in Figure 1A).

#### Stimulus

In the visual test, a red stationary circle was displayed at the center of the screen. The diameter of the circle varied depending on the distance being measured, with a diameter of 15.15 mm at 3 m, and a diameter of 11.65 mm at 0.77 m and 0.40 m. A white horizontal line, 0.47 mm in width, was situated under the red circle. A white circle with the same size as the red one, was movable by the input of the mouse from the subjects.

#### Test procedure

Subjects viewed the display binocularly with their best corrections in a dim room. The spectacle for examination was worn throughout the entire measurement. Subjects were shown a red vertical line on the right eye, because through the Maddox rod (on the right eye of the glass in Figure 1A), a red fixed circle would become a red vertical line. Subjects were instructed to move the white circle to the cross point of the red vertical line and the white horizontal line (shown in Figure 1B), and to subsequently respond with the click using the mouse. After the click, the response would be recorded and the white point would move elsewhere randomly so that the positional bias could be removed. Subjects were then instructed to find the cross point and click again. The test was performed three times and the average was transformed into prism diopter and was outputted in the forms of the mean and standard deviation. The tests at distance of 3 m, 0.77 m and 0.40 m were performed in order. During the 0.40-meter test, subjects were asked to keep the sentence above clear to keep accommodation stable.

### Prism-neutralized objective cover test (POCT)

#### Equipment

A prism bar with 2^△^ increment from 0^△^ to 20^△^ and 5^△^ increment from 25^△^ to 40^△^ was used in measurement.

#### Stimulus

The fixating targets used in the study were 20/30 letters, with their size determined by the respective distance. However, at distances of 0.77 m and 0.40 m, the same letters were used as the differences in size were negligible (less than 1 mm).

#### Test procedure

Subjects were examined with corrected refractive errors, and they were told to keep the target letter clear. A prism bar was held no further than 1 cm from the right eye while the alternate cover test was performed. The test procedure followed that described by Johns et al. (2004), and the endpoint was determined as the midpoint of the first neutral and reversal points. Subjects repeated the test three times and the mean of their results was recorded as their final heterophoria value.

#### Study design

In light of the study by Bland and Altman (Martin Bland and Altman, 1986), we thought the best way to assess the repeatability of an instrument was to take several measurements in a series of subjects. In the first part of the study, the precision of the CHT was determined by inter- and intra-examiner repeatability. The first session was designed to determine inter-examiner repeatability, which refers to the reliability (agreement) of measurements between two different examiners. Three valid CHT tests were performed and considered independent since the white point was moved randomly. The time required to acquire three measurements was approximately 1 min. In the second session, intra-examiner repeatability was evaluated, which assesses the reliability of measurements between two sessions by the same examiner. The time interval between these two sessions varied from two to 7 days.

During the second part of the study, the agreement between the CHT and POCT was analyzed. Both tests were performed for each subject in the first session, and the order was randomized. CHT was administered by two novice optometrists, while POCT was conducted by an experienced optometrist who had more than 5 years of clinical experience. In the second session, all the examiners were blinded to the previous test results.

### Statistical analysis

All data were analyzed using the SPSS for Windows software (version 22.0, SPSS, Inc.). A repeated-measures analysis of variance (ANOVA) with two factors (examiner and session) was conducted to examine the overall effects. *p* < 0.05 means statistical significance.

The repeatability of CHT between two examiners and two sessions, and the agreement between CHT and POCT were determined using the Bland–Altman method (Martin Bland and Altman, 1986; Zadnik et al., 1992). Comparison between CHT and POCT was analyzed with paired Student *t* tests. The variables used to evaluate were the mean difference (MD), the standard deviation of difference (SD), limits of agreement between two tests, the coefficient of repeatability (COR = ±1.96*SD), and Spearman correlation ratios between two tests.

Romano and von Noorden reported that the smallest eye movement detectable in a cover test is 2^△^ (Romano and Von Noorden, 1971). In clinical practice, Rainey suggested that a difference of ±4^△^ or less between the 95% limits of agreement would be clinically acceptable (Schroeder et al., 1996). Therefore, a ± 4^△^ difference should be considered as the standard for evaluating clinical agreement. A one-tailed Student t-test was used to compare the absolute difference between POCT and CHT and 4^△^. *p* < 0.05 means statistical significance.

## Results

A total of 103 subjects aged 20 to 48 (mean, 27.37 years; SD, 5.15 years) were enrolled in the study. The mean (SD) of spherical equivalent refractive errors for subjects were − 3.47 (1.48) D.

### Repeatability of CHT

The heterophoria measurements obtained by two examiners on two different days using the CHT did not show any statistical significance at three different distances. The results of the repeated-measures analysis of variance are presented in Table 1, and the heterophoria measurements were not significantly different between the two examiners at three different distances (*p* > 0.05). Moreover, there was no statistically significant difference in heterophoria measurements between the two sessions (*p* > 0.05), and the interactions between examiners and sessions were not significant (*p* > 0.05).

**Table 1:** Statistic description of repeated measurements of CHT, *n* = 103.

Test distance	3 m	0.77 m	0.40 m
E1 S1	−0.47(−2.73, 0.02)	−1.23(−4.90, −0.12)	−3.07(−7.71, −0.18)
E2 S1	−0.42(−2.67, 0.04)	−1.37(−4.79, −0.01)	−2.74(−8.71, −0.21)
E1 S2	−0.95(−2.61, 0.05)	−1.36(−4.64, −0.05)	−3.30(−8.19, −0.17)
E2 S2	−0.82(−2.67, 0.03)	−1.12(−4.96, 0.00)	−3.28(−7.88, −0.09)
Examiner	*F* = 1.974, *p* = 0.163	*F* = 0.044, *p* = 0.835	*F* = 1.890, *p* = 0.172
Session	*F* = 0.312, *p* = 0.577	*F* = 0.786, *p* = 0.377	*F* = 0.029, *p* = 0.866
Examiner* Sessions	*F* = 1.476, *p* = 0.227	*F* = 1.009, *p* = 0.317	*F* = 0.317, *p* = 0.575

E1 means examiner 1; E2 means examiner 2; S1 means session 1; S2 means session 2; Examiner means repeated-measures analysis of variance based on the factor of examiner; Session means repeated-measures analysis of variance based on the factor of sessions; Examiner*Sessions means repeated-measures analysis of variance based on the interaction factor of the examiner and the session. The data are presented as medians and quartiles.

**Figure 2:**
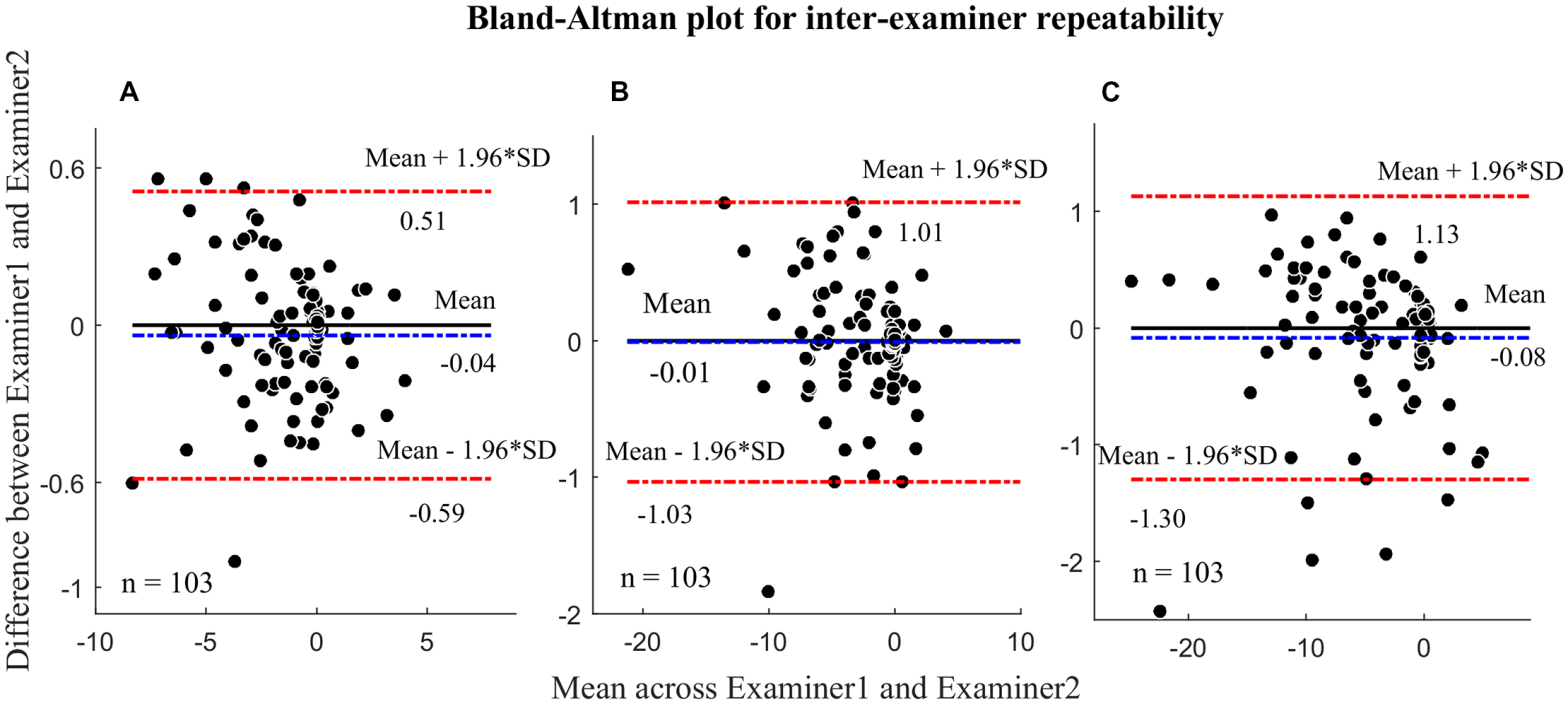
Bland-Altman plots illustrating the inter-examiner repeatability. **(A)** Heterophoria measured at 3 meters; **(B)** Heterophoria measured at 0.77 meters; **(C)** Heterophoria measured at 0.40 meters. The unit in the figure is prism diopters (Δ).

**Figure 3:**
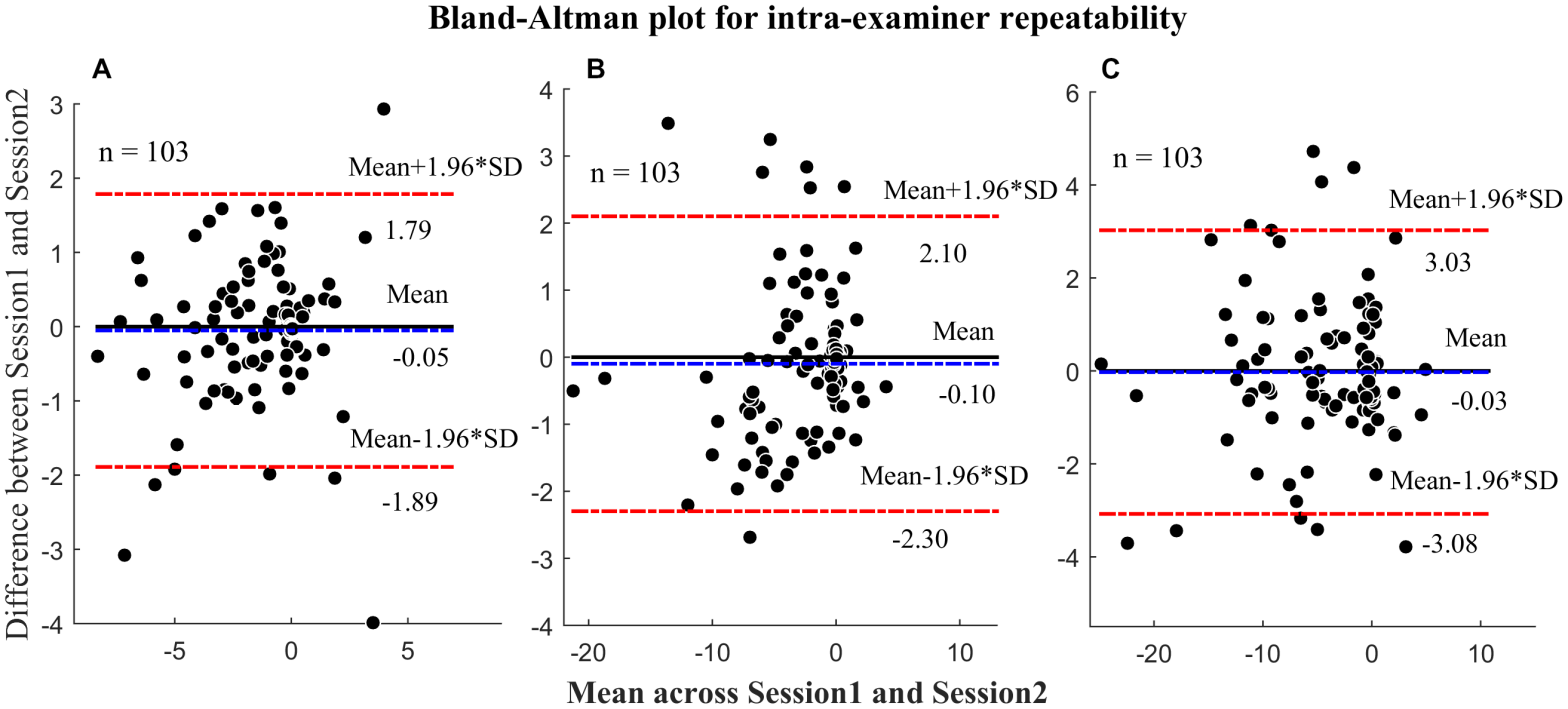
Bland-Altman plots illustrating the intra-examiner repeatability. **(A)** Heterophoria measured at 3 meters; **(B)** Heterophoria measured at 0.77 meters; **(C)** Heterophoria measured at 0.40 meters. The unit in the figure is prism diopters (Δ).

Inter- and intra-examiner repeatability coefficients for the different distance tests as well as Bland–Altman plots are shown in Figures 2, 3, respectively. The MD (COR) of inter-examiner repeatability were − 0.04^△^ (±0.55^△^) at 3 m, −0.01^△^ (±1.02^△^) at 0.77 m, and − 0.08^△^ (±1.21^△^) at 0.40 m. The MD (COR) of intra-examiner repeatability were − 0.05 (±1.83^△^) at 3 m, −0.10 (±2.19^△^) at 0.77 m, −0.03 (±3.04^△^) at 0.40 m. The MD were smaller than 2^△^ and COR was smaller than 4^△^, indicating that the results were within a clinically acceptable range.

### Agreement between CHT and POCT

**Figure 4:**
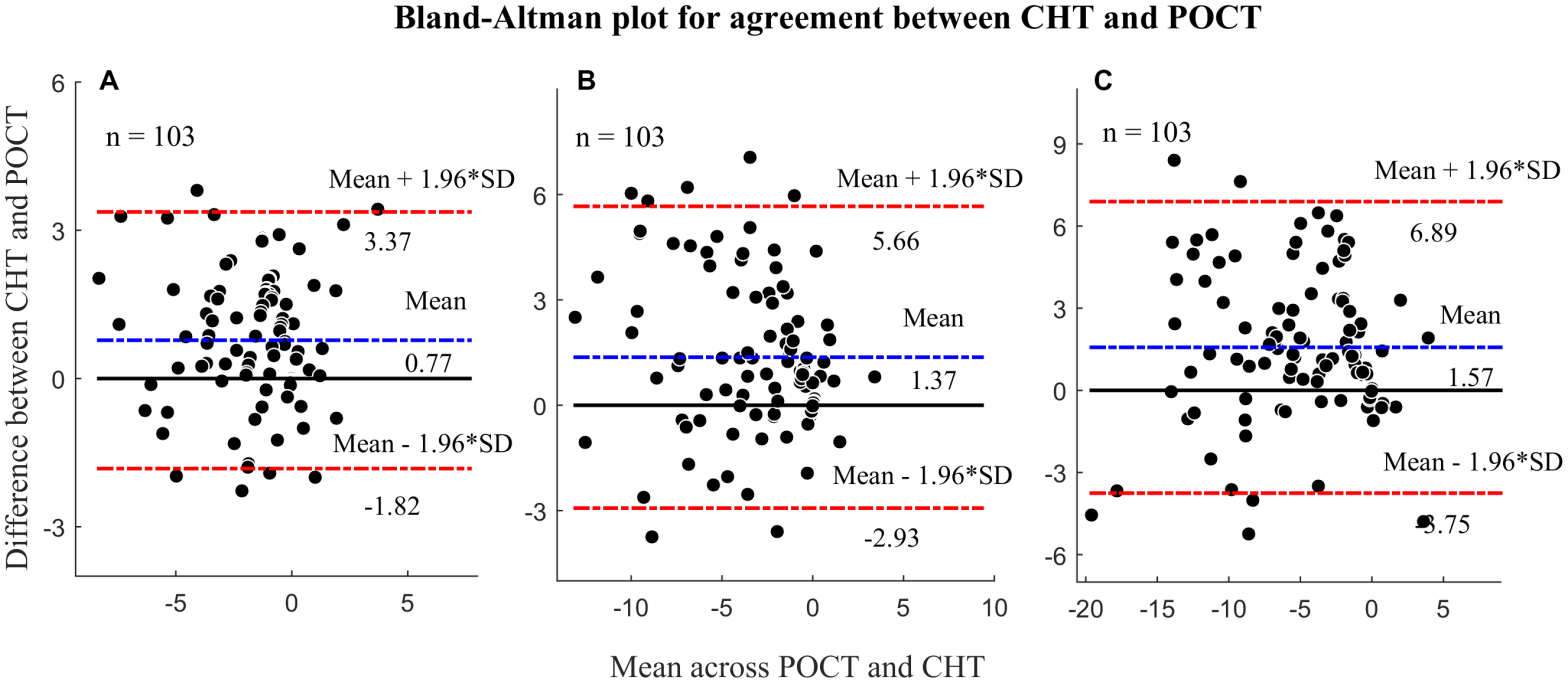
Bland-Altman plots illustrating the agreement between CHT and POCT. **(A)** Heterophoria measured at 3 meters; **(B)** Heterophoria measured at 0.77 meters; **(C)** Heterophoria measured at 0.40 meters. The unit in the figure is prism diopters (Δ). *p* < 0.025 means statistical significance.

**Figure 5:**
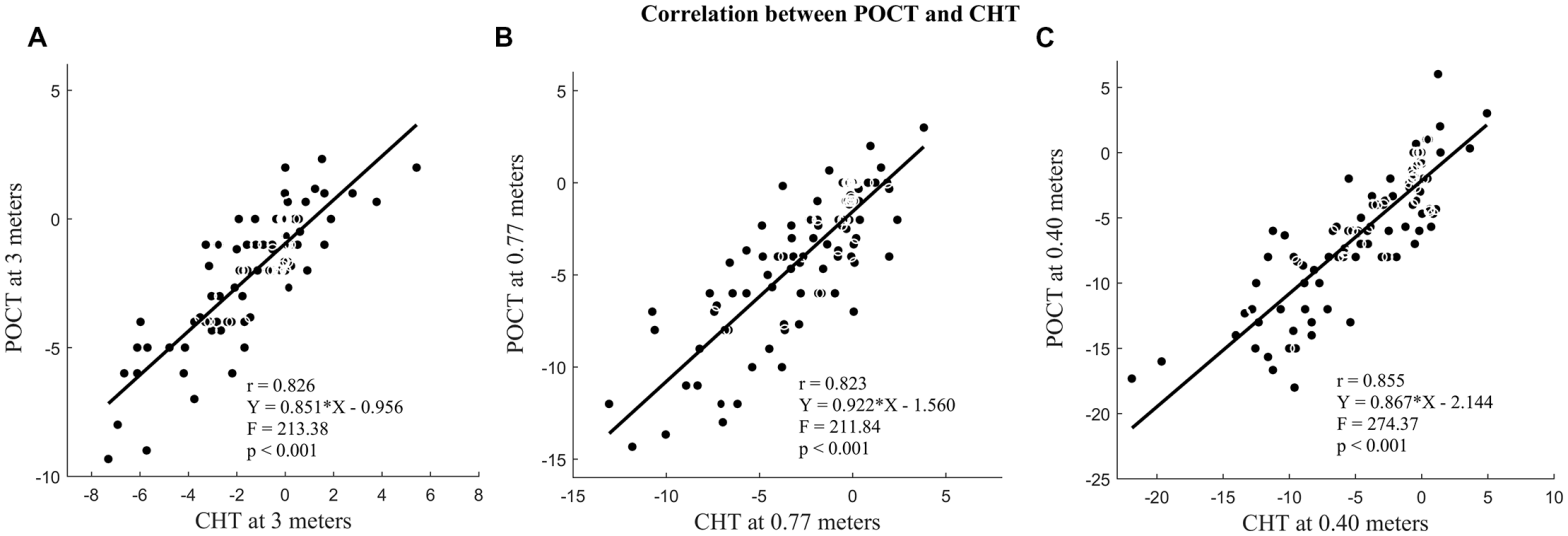
Correlation between POCT and CHT. **(A)** Heterophoria measured at 3 meters; **(B)** Heterophoria measured at 0.77 meters; **(C)** Heterophoria measured at 0.40 meters. The unit in the figure is prism diopters (Δ).

The measurements of heterophoria using CHT and POCT showed a significant difference, but the difference was within a clinically acceptable range. Table 2 displays the description and agreement of heterophoria measurements by CHT and POCT, and Figure 4 shows the Bland–Altman plots. The differences between POCT and CHT at three different measurements were found to be significantly different (*p* < 0.001). However, there was a large correlation between CHT and POCT at 3 m (*r* = 0.826, *p* < 0.001), 0.77 m (*r* = 0.823, *p* < 0.001), and 0.40 m (*r* = 0.855, *p* < 0.001), as shown in Figure 5. Meanwhile, at 3 m, all differences between CHT and POCT were within ±4 ^△^. At 0.77 m, 87 of 103 (84.47%) differences were within ±4^△^. At 0.40 m, 80 of 103 (77.67%) differences were within ±4^△^. The absolute difference between POCT and CHT was found to be smaller than 4^△^ at three different distances (*p* < 0.001). Additionally, the absolute difference between POCT and CHT was smaller than 2^△^ at 3 m (*t* = −8.52, *p* < 0.001).

**Table 2:** Description of statistics and agreement of heterophoria measured by POCT and CHT, *n* = 103.

Test distance	3 m	0.77 m	0.40 m
CHT	−0.52(−2.22, 0.02)	−1.27(−4.18, −0.08)	−3.40(−7.41, −0.35)
POCT	−2.00(−3.83, −0.25)	−3.00(−6.00, −1.00)	−5.67(−8.00, −2.00)
CHT - POCT	0.77 ± 1.32 *t*_1_ = 5.93, *p*_1_ < 0.001	1.37 ± 2.18 *t*_1_ = 6.33, *p*_1_ < 0.001	1.57 ± 2.70 *t*_1_ = 5.87, *p*_1_ < 0.001
| CHT – POCT |	1.20 ± 0.95 *t*_2_ = −8.52, *p*_2_ < 0.001	1.93 ± 1.71 *t*_2_ = −12.28, *p*_2_ < 0.001	2.41 ± 2.00 *t*_2_ = −8.07, *p*_2_ < 0.001
COR	±2.59	±4.27	±5. 30

*t*_1_ and *p*_1_ denote the results of paired student *t* tests between CHT and POCT. *t*_2_ and *p*_2_ denote the results of student *t* tests between | CHT – POCT | and 4^△^, but the *t*_2_ and *p*_2_ at 3 m mean the result of student *t* tests between | CHT – POCT | and 2^△^. The data are presented as medians and quartiles. *p* < 0.025 means statistical significance.

## Discussion

In this study, we developed and validated the CHT by conducting repeated measurements with two different examiners across two different sessions. Our results demonstrate that the CHT has excellent inter- and intra-examiner repeatability. We then analyzed its repeatability and agreement with POCT. There was a strong correlation between the two methods, and the absolute difference between them was within a clinically acceptable range. Thus, the CHT could be considered as interchangeable with POCT. While POCT is generally considered reliable, its accuracy depends heavily on the experience of the examiner. In contrast, CHT demonstrated good reliability even with novice examiners. Additionally, CHT is a more convenient and efficient method of measuring heterophoria, which making it particularly suitable for use in settings such as outpatient triage and community screenings. Given these advantages, CHT has significant potential for improving the accuracy and efficiency of heterophoria measurements in clinical and community settings.

### Repeatability of CHT

The results presented in Table 1 indicate excellent repeatability of the CHT test, with no significant differences observed between examiners or sessions. Additionally, the COR values were all less than 4^△^, which is an acceptable range for clinical measurements (Schroeder et al., 1996). This is due to the fact that the design of the CHT is based on TH, which is known for its high reliability in measurement (Cebrian et al., 2014). TH and POCT has been widely used for measuring ocular deviations (Johns et al., 2004; Cebrian et al., 2014), and the inter-examiner COR were 1.43^△^ and ± 1.65^△^ for TH and POCT at distance, respectively. Our results showed the COR of CHT at distance was only 0.55^△^, which is better than TH and POCT. Similarly, the intra-examiner repeatability of CHT (±1.83^△^) was found to be comparable to that of TH (±1.28^△^) and POCT (±1.51^△^). These results were consistent at near as well. Thus, based on our findings, CHT demonstrates reliable repeatability at both 3 m and 0.40 m. Notably, there have been no previous investigations into the inter- and intra-examiner repeatability of heterophoria measurement at a middle distance (0.77 m). In this study, we found that the inter- and intra-examiner COR values at this distance were ± 1.02^△^ and ± 2.19^△^ respectively, both of which were less than 4^△^. These results indicate that the CHT exhibits good repeatability across near, middle, and far distances, making it comparable to TH and POCT methods.

### Agreement

The results showed a strong correlation between CHT and POCT measurements, despite the CHT indicating greater esophoria compared to POCT. The significant differences between the POCT and other methods were also reported in other studies, for example, Cebrian et al. measured distance heterophoria using TH, MR, von Graefe (VG) method and POCT, and they found significant differences between any of the tests and POCT (Cebrian et al., 2014). Compared with Cebrian’s study, the difference between CHT and POCT was comparable (0.77^△^ for CHT versus 0.63^△^, 0.44^△^, and 0.68^△^ TH, MR, and VG respectively). In our study, the mean absolute differences of two measurements at three different distances were smaller than 4^△^, as mentioned above, which is a clinical acceptable range. Additionally, the mean absolute difference at 3 m was less than the resolution of POCT (2^△^).

Our results indicated that the agreement between CHT and POCT is worse at near than far distance. Many studies concluded the variation of near heterophoria is much larger than far heterophoria (Schroeder et al., 1996; Canto-Cerdan et al., 2018). One of the reasons is accommodation. Canto-Cerdan and colleagues (Canto-Cerdan et al., 2018) analyzed the agreement between POCT and VG measurements for both non-presbyopic and presbyopic subjects. They found that the results for presbyopes showed better agreement, indicating that accommodation may play a role in phoria measurements. During the CHT test, subjects were instructed to maintain the sentences on the screen clear, which helped stabilize their accommodation at a constant level. Moreover, a previous clinical study demonstrated that heterophoria measurement with the trial frame exhibited better repeatability than with the phoropter (Casillas and Rosenfield, 2006). The reason for this could be attributed to peripheral fusion, which can induce fusional vergence when retinal non-corresponding points are active spontaneously (Burian, 1939). Measuring with the trial frame was performed in free space and therefore provided a wide visual field, whereas the phoropter would restrict a relatively larger visual field. It is logical to infer that the presence of the peripheral stimuli, even under dissociate conditions, induced peripheral fusion when measuring heterophoria with trial frame. As a result, measuring heterophoria with the trial frame showed better repeatability than with the phoropter. Additionally, the previous study also demonstrated that TH had better repeatability than VG and MR at distance (Casillas and Rosenfield, 2006). Since the CHT combines the advantages of the trial frame and TH, it offers even greater repeatability.

## Conclusion

CHT exhibits good test–retest repeatability and measurement agreement. Since CHT minimizes the measurement variances that can originate from examiners, results from CHT do not create external sources of measurement errors. Additionally, CHT is more flexible and user-friendly during measurement. Therefore, we believe that CHT can be considered interchangeable with POCT and is worthy of widespread use in clinical settings.

## Data availability statement

The original contributions presented in the study are included in the article/supplementary material, further inquiries can be directed to the corresponding author.

## Ethics statement

The studies involving human participants were reviewed and approved by the Ethics Committee of the Eye Hospital of Wenzhou Medical University. The patients/participants provided their written informed consent to participate in this study.

## Author contributions

YW, FuZ, and ES contributed to conception and design of the study. FuZ performed the experiments, analyzed the data, and drafted the manuscript. FeZ revised the manuscript. All authors have reviewed and approved the final version for publication.

## Conflict of interest

The authors declare that the research was conducted in the absence of any commercial or financial relationships that could be construed as a potential conflict of interest.

## Publisher’s note

All claims expressed in this article are solely those of the authors and do not necessarily represent those of their affiliated organizations, or those of the publisher, the editors and the reviewers. Any product that may be evaluated in this article, or claim that may be made by its manufacturer, is not guaranteed or endorsed by the publisher.
